# Corrigendum to “Systematic post-translational modification genome wide identifies therapeutic targets for Alzheimer’s disease: evidence from multi-cohort analysis” [J Prev Alzheimer Dis, 13 (2026) 100422]

**DOI:** 10.1016/j.tjpad.2026.100593

**Published:** 2026-05-15

**Authors:** Xiaoming Wang, Yuancheng Liu, Juncai Fu, Yizhao Li, Mengying Zhao, Qing Tian

**Affiliations:** a Department of Pathophysiology, School of Basic Medicine, Key Laboratory of Neurological Diseases of Hubei Province and National Education Ministry, Tongji Medical College, Huazhong University of Science and Technology, Wuhan, China; b Innovation Center for Brain Medical Sciences of the Ministry of Education, Huazhong University of Science and Technology, Wuhan 430030, China

The Acknowledgments section of this article should have been published as below.

**Acknowledgments**

This work was supported by grants from the National Natural Science Foundation of China (82071478) and Key project of Natural Science Foundation of Hubei Province (2023 AFD123). We also thank Medical sub-center of Analytical and Testing Center, Huazhong University of Science and Technology, for the data analysis of this work.

The authors would like to apologise for any inconvenience caused.

